# Current status and influencing factors of early delayed neurocognitive recovery in older adult patients after major orthopedic surgery under general anesthesia: a cohort study

**DOI:** 10.3389/fpubh.2026.1887947

**Published:** 2026-08-03

**Authors:** Siyu Yan, Dan Zhou, Yinghao Zhou, Lei Song, Xiaolin Xu, Jiamin Wang, Lin Zhao

**Affiliations:** 1 School of Nursing, Qingdao University, Qingdao, China; 2 Department of Nursing, Affiliated Hospital of Qingdao University, Qingdao, China; 3 Department of Spine Surgery, Affiliated Hospital of Qingdao University, Qingdao, China; 4 Department of Hospital Infection Control, Affiliated Hospital of Qingdao University, Qingdao, China; 5 Department of Anesthesiology, Affiliated Hospital of Qingdao University, Qingdao, China

**Keywords:** cohort study, dNCR, older adults, influencing factors, orthopedic surgery

## Abstract

**Objective:**

To investigate the current status of early delayed neurocognitive recovery (dNCR) in older adult patients after major orthopedic surgery in China and analyze its influencing factors, providing a reference for early intervention by clinical workers.

**Design:**

Observational cohort study.

**Methods:**

A convenience sampling method was used to select patients aged 60 years or older who underwent major orthopedic surgery under general anesthesia at a tertiary hospital in Qingdao, Shandong Province, from September 2025 to June 2026. A general information questionnaire, depression scale, frailty scale, nutrition scale, and sleep quality scale were used to collect data 1 day before surgery. The Beijing version of the Montreal Cognitive Assessment (MoCA) was administered 1 day before surgery and on postoperative day 7 or before discharge (whichever occurred first). Variables with *p* < 0.2 in the univariate analysis were entered into the binary logistic regression model to identify factors influencing dNCR.

**Results:**

A total of 505 patients were included, of whom 155 (30.69%) developed dNCR. Regression analysis showed that age, sleep disorder, anesthesia time, and stroke history were independent risk factors for postoperative dNCR, and the MoCA score was a protective factor (*p* < 0.05).

**Conclusion:**

After major orthopedic surgery, dNCR is relatively common in older adult patients. Clinical workers should strengthen early preoperative screening, identify high-risk patients, and provide targeted interventions and cognitive training, so as to improve patient outcomes.

## 1 Introduction

Major orthopedic surgery, including total knee arthroplasty (TKA), total hip arthroplasty (THA), and hip fracture (HF) surgery, is the mainstay of treatment for end-stage osteoarthritis (OA) (1). OA is a chronic degenerative disease of articular cartilage that most commonly affects the knee joint, followed by the hand and hip joints (2). As the disease progresses, pathological changes gradually involve bone tissue and bone marrow, worsening pain and accelerating the disease process (3). Because OA often affects the lower limb joints, leading to joint capsule stiffness, ligament laxity, and muscle weakness around the joints, it has become one of the leading causes of disability among the global population aged 60 and older (4, 5). The prevalence of OA is closely related to advanced age and long-term joint loading, and its global burden is substantial and continues to rise. Over the past 20 years, the number of people with OA increased by 134.48%, with prevalence influenced by regional economic levels, and higher in developed regions than in less developed ones (6). Among Chinese patients aged 60 and older, the prevalence of OA is as high as 43.7% (7). Due to global aging, the number of patients with OA is expected to reach 1 billion by 2050 (8).

Currently, there is a lack of effective drugs that can slow the progression of OA, and treatment largely relies on major orthopedic surgery such as joint replacement to improve patients’ quality of life (9). However, due to various factors such as surgical trauma, anesthesia suppression, and inflammatory response, older adult patients often experience a decline in memory, attention, executive function, and other cognitive domains within 30 days after major orthopedic surgery, compared with their preoperative levels. This condition is termed delayed neurocognitive recovery (dNCR) (10, 11). These cognitive changes are subtle and not directly observable, requiring neuropsychological testing to identify the alterations in cognitive function. Although dNCR emphasizes its reversibility and recovery potential, early prevention and intervention remain critically important for these patients. Studies have indicated that without timely intervention, patients with postoperative dNCR have up to a 70% probability of progressing to dementia, which in turn prolongs hospital stay and increases the burden on family care and the economy (12). However, existing studies have mainly focused on patients undergoing a single procedure, such as TKA or THA, while specific research on the broader population of patients with OA remains relatively limited. Since TKA, THA, and non-emergency HF surgery share the same underlying pathology, namely OA, this study aims to investigate the current status and influencing factors of postoperative dNCR in this OA patient population, thereby providing a broader clinical perspective.

Based on the PECO framework, this prospective cohort study was designed as follows: Population (P) comprised older adult patients (aged ≥60 years) who underwent major orthopedic surgery under general anesthesia, including TKA, THA, and non-emergency HF surgery. Exposure (E) included potential influencing factors for early dNCR, covering sociodemographic data, disease-related data, laboratory indicators, and surgery-related factors. Comparison (C) evaluated the differences in all potential influencing factors between patients who developed dNCR and those who did not. Outcome (O) was the occurrence of early dNCR at postoperative day 7 or before discharge (whichever came first), diagnosed according to the Evered (13) consensus criteria.

## 2 Methods

### 2.1 Study subjects

Using convenience sampling, older adult patients aged 60 years and above who underwent major orthopedic surgery under general anesthesia in a tertiary hospital in Qingdao, Shandong Province, from September 2025 to June 2026 were selected. Inclusion criteria: (1) age ≥ 60 years; (2) undergoing major orthopedic surgery under general anesthesia, including TKA, THA, or HF surgery; (3) no preoperative cognitive impairment, defined as a preoperative Montreal Cognitive Assessment Beijing Version (MoCA) score of >13 for illiterate subjects, >19 for those with primary school education, and >24 for those with junior high school education or above; (4) on-emergency surgery; and (5) not admitted to the ICU after surgery. Exclusion criteria: (1) presence of severe hearing or visual impairment; (2) development of overt consciousness disturbance after surgery, such as hallucination, lethargy, or agitation; and (3) refusal to participate in this study.

### 2.2 Sample size calculation

Sample size was calculated based on the method for multivariate analysis, estimated as 5 to 10 times the number of independent variables (14). This study included 27 independent variables, so the required number of positive samples was 135–270 cases, of which 155 were ultimately included.

### 2.3 Research tools

#### 2.3.1 General information questionnaire

The questionnaire was developed by the researchers based on the study purpose and a literature review. It includes: (1) Socio-demographic data: age, gender, body mass index (BMI), type of surgery, smoking, alcohol use, and education level. (2) Disease-related data: diabetes, hypertension, stroke history, and coronary heart disease. (3) Laboratory indicators: neutrophil-to-lymphocyte ratio (NLR), albumin (ALB), C-reactive protein (CRP), alanine aminotransferase (ALT), aspartate aminotransferase (AST), creatinine (Cr), and glomerular filtration rate (GFR). (4) Surgery-related factors: operative time, anesthesia time, and intraoperative blood loss.

#### 2.3.2 Beijing version of the Montreal Cognitive Assessment

The Beijing version of the Montreal Cognitive Assessment (MoCA) was developed by Nasreddine et al. (15), was translated and revised by Wang et al. (16), and has been widely used to assess the cognitive status of study participants. The scale evaluates seven cognitive domains, including visuospatial and executive function, naming, attention, language, abstract reasoning, delayed recall, and orientation. The total score ranges from 0 to 30. The scale has different cutoff points based on education level: patients with illiteracy ≤13 points, primary school education ≤19 points, and junior high school education or above ≤24 points are considered to have cognitive impairment (17). In addition, an extra point is awarded to participants with ≤12 years of formal education (18). A higher score indicates better cognitive function. The time window for dNCR extends from immediately after surgery to 30 days postoperatively. Clinically, this condition manifests as mild cognitive changes that are not readily identifiable through routine observation and usually require neuropsychological testing for diagnosis. In the present study, the MoCA was administered 1 day before surgery and again at postoperative day 7 or before discharge, whichever occurred first, to capture the early phase of dNCR. A decline of at least 1 standard deviation in the postoperative MoCA score relative to the preoperative MoCA score of the study population was defined as dNCR (13). The Cronbach’s *α* coefficient of the scale is 0.850 (19).

#### 2.3.3 Patient health questionnaire-9

The Patient health questionnaire-9 (PHQ-9) was developed by Kroenke et al. (20), was translated and revised by Bian et al. (21), and has been widely used to assess the depression level of study participants. The scale consists of 9 items and uses a 4-point scoring method. The total score ranges from 0 to 27, which is divided into five levels: 0–4 for no depression, 5–9 for mild depression, 10–14 for moderate depression, 15–19 for moderately severe depression, and 20–27 for severe depression. The Cronbach’s *α* coefficient of the scale is 0.910 (13).

#### 2.3.4 Frailty scale

The Frailty scale (FRAIL) was proposed by experts of the International Nutrition, Health and Aging Working Group (22), and was translated by Wei et al. (23). It comprises 5 items, each assigned a score of 1 point, yielding a total score ranging from 0 to 5. A score of 0 indicates no frailty, a score <3 indicates pre-frailty, and a score ≥3 indicates frailty. The Cronbach’s *α* coefficient of the scale is 0.826.

#### 2.3.5 Nutritional risk screening scale-2002

The Nutritional risk screening scale-2002 (NRS-2002) was developed by Kondrup et al. (24) and recommended by the Chinese Society of Parenteral and Enteral Nutrition (25). The scale includes three dimensions: disease status, nutritional status, and age. Instead of summing individual item scores within each dimension, only the highest score of each dimension is adopted. The total score ranges from 0 to 7, and a score of ≥3 indicates nutritional risk.

#### 2.3.6 Pittsburgh sleep quality index

The Pittsburgh sleep quality index (PSQI) was developed by Buysse et al. (26) and was translated and revised by Liu et al. (27). The scale consists of 18 items and covers 7 dimensions: sleep quality, sleep latency, sleep duration, sleep efficiency, sleep disturbance, use of sleeping medication, and daytime dysfunction. Each dimension is scored from 0 to 3, and the total score ranges from 0 to 21. A total score below 7 indicates good sleep quality, 7–11 indicates poor sleep quality, and above 11 indicates very poor sleep quality. The Cronbach’s *α* coefficient of the scale is 0.842.

#### 2.3.7 American society of anesthesiologists classification

The American society of anesthesiologists classification (ASA) was developed by Sankar et al. (28). The scale divides patients’ health status into six levels. Level 1 indicates that the patient is healthy, and level 6 indicates that the patient is brain dead. Higher ASA levels correspond to poorer physical condition and elevated perioperative anesthetic and surgical risks. The ASA classification exhibits moderate inter-rater reliability in clinical practice, with a Kappa coefficient of 0.61, and plays an important role in predicting patients’ postoperative outcomes (28).

### 2.4 Data collection and quality control

Before data collection, all investigators received standardized training and assessment. The training included the MoCA cognitive assessment and standardized data collection procedures. Standardized verbal instructions were used to explain the study’s purpose, significance, and methods to patients. After obtaining informed consent, the questionnaire survey was conducted by trained investigators who were blinded to patients’ intraoperative data. Following data collection, two independent researchers double-entered the data into a computer; any inconsistencies identified were verified and corrected promptly.

### 2.5 Statistical analysis

Statistical analysis was performed using SPSS 26.0 and R 4.5.2. Normally distributed continuous data were presented as $\bar{x}$ ±*s*, and comparisons between two groups were conducted using the independent samples *t*-test. Non-normally distributed continuous data were presented as median (IQR), with group comparisons performed using the Wilcoxon rank sum test. Categorical variables were expressed as frequencies (*n*) and percentages (%) and compared using the χ^2^ test or Fisher’s exact test. Missing postoperative MoCA data in patients lost to follow-up were handled using multiple imputation by chained equations (MICE), with predictive mean matching (PMM) applied as the imputation method. Variables with *p* < 0.2 in univariate analysis were entered into a multivariable logistic regression model, and results were pooled using Rubin’s rules. Sensitivity analysis comparing complete-case analysis with multiple imputation was performed to assess the robustness of the findings. Receiver operating characteristic (ROC) curves were plotted and the area under the curve (AUC) was calculated; comparisons between models were performed using the DeLong test. A two-sided *p* < 0.05 was considered statistically significant.

## 3 Results

### 3.1 Patient demographic and clinical characteristics

**Figure 1:**
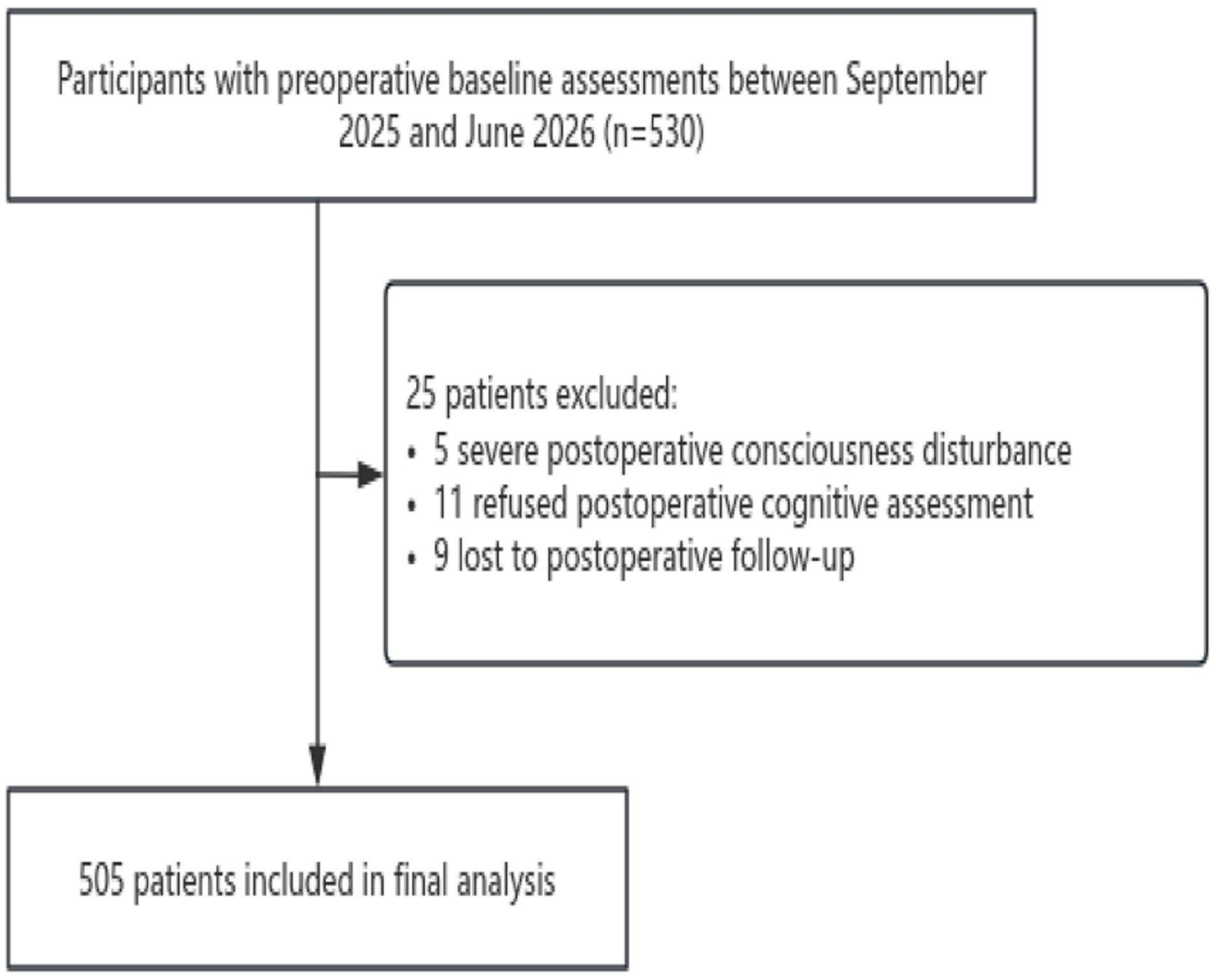
Flowchart of study participants.

A total of 505 older adult patients undergoing orthopedic surgery under general anesthesia were finally enrolled in this study. Among them, 155 patients (30.69%) developed dNCR, and 350 patients (69.31%) did not. The flow chart of participant selection is shown in Figure 1. Regarding the type of surgery: 351 patients (69.50%) received TKA, 94 patients (18.61%) received THA, and 60 patients (11.88%) received HF surgery. In the dNCR group, there were 28 males (18.06%) and 127 females (81.94%); in the non-dNCR group, there were 96 males (27.35%) and 254 females (72.36%). Clinical data were compared between the two groups using univariate analysis (see Table 1).

**Table 1:** Comparison of general data between two groups (*n* = 505).

Variables	Non- dNCR	dNCR	*Z/χ^2^*	*P*
Age [years, median (IQR)]	68.00 (64.00, 71.00)	74.00 (70.00, 78.00)	−10.009	<0.001*
Gender (*n*, %)			5.085	0.024*
Male	96 (27.4%)	19 (19.6%)		
Female	254 (72.6%)	78 (80.4%)		
BMI	25.95 (23.7, 28.68)	25.7 (22.46, 28.82)	−0.309	0.757
Type of surgery			16.473	<0.001*
TKA	255 (72.9%)	40 (15.9%)		
THA	67 (19.1%)	19 (19.6%)		
HF	28 (8.0%)			
Smoking (*n*, %)			0.325	0.569
No	315 (90.0%)	92 (94.8%)		
Yes	35 (10.0%)	5 (5.2%)		
Alcohol (*n*, %)			0.747	0.388
No	314 (89.7%)	83 (85.5%)		
Yes	36 (10.3%)	14 (14.4%)		
Education level (*n*, %)			35.271	<0.001*
Illiteracy	34 (9.7%)	29 (29.9%)		
Primary school	94 (26.9%)	41 (42.3%)		
Junior high school and above	222 (63.4%)	27 (27.8%)		
Diabetes (*n*, %)			0.755	0.385
No	287 (82.0%)	77 (79.4%)		
Yes	63 (18.0%)	20 (20.6%)		
Hypertension (*n*, %)			0.045	0.831
No	119 (54.6%)	54 (55.7%)		
Yes	159 (45.4%)	43 (44.3%)		
Stroke history (*n*, %)			18.013	<0.001*
No	338 (96.6%)	84 (86.6%)		
Yes	12 (3.4%)	13 (13.4%)		
Coronary heart disease (*n*, %)			5.936	0.015*
No	307 (87.7%)	78 (80.4%)		
Yes	43 (12.3%)	19 (19.6%)		
ASA grade (*n*, %)			4.906	0.027*
II	77 (22.0%)	13 (13.4%)		
III	273 (78.0%)	84 (86.6%)		
NLR [109/L, median (IQR)]	2.05 (1.51, 2.96)	2.21 (1.56, 3.65)	−1.026	0.305
ALB[g/L, median (IQR)]	43.00 (40.00, 44.80)	42.80 (39.50, 45.00)	−1.848	0.065*
CRP [mg/L, median (IQR)]	0.75 (0.50, 1.89)	0.56 (0.50, 5.33)	−1.864	0.062*
ALT [U/L, median (IQR)]	16.00 (12.75, 22.00)	15.00 (11.00, 20.00)	−3.383	0.001*
AST [U/L, median (IQR)]	19.00 (16.00, 23.00)	19.00 (16.00, 23.00)	−0.797	0.425
Cr [μmol/L, median (IQR)]	53.00 (45.00, 63.00)	55.00 (49.00, 65.00)	−0.608	0.543
GFR [ml/min, median (IQR)]	95.76 (89.96, 101.73)	89.89 (84.45, 95.91)	−5.836	<0.001*
Intraoperative Blood Loss [ml, median (IQR)]	50.00 (50.00, 200.00)	50.00 (50.00, 200.00)	−2.212	0.034*
Operation time [min, median (IQR)]	80.00 (65.00, 95.00)	110.00 (85.00, 120.00)	−6.545	<0.001*
Anesthesia time [min, median (IQR)]	140.00 (125.00, 160.00)	170.00 (152.50, 190.00)	−6.868	<0.001*
MOCA [score, median (IQR)]	25.00 (22.00, 26.00)	21.00 (17.00, 25.00)	−7.449	<0.001*
Weakened (*n*, %)			23.605	<0.001*
No	37 (93.4%)	76 (78.4%)		
Yes	23 (6.6%)	21 (21.6%)		
Depression (*n*, %)			31.834	<0.001*
No	329 (94.0%)	65 (67.0%)		
Yes	21 (6.0%)	32 (33.0%)		
Nutritional risk (*n*, %)			8.119	0.004*
No	343 (98.0%)	88 (90.7%)		
Yes	7 (2.0%)	9 (9.3%)		
Sleep disorder (*n*, %)			17.604	<0.001*
No	260 (74.3%)	51 (52.6%)		
Yes	90 (25.7%)	46 (47.4%)		

*: *P* < 0.2.

### 3.2 Multicollinearity check

Before binary logistic regression analysis, multicollinearity was tested for variables with *p* < 0.2 in the univariate analysis. Due to inherent correlations among variables, a stricter threshold was adopted such that a variance inflation factor (VIF) < 3 indicated no multicollinearity and allowed logistic regression analysis (29). Education, MoCA score, operation time, and anesthesia time showed collinearity. Therefore, based on their research value and correlation with postoperative cognitive function, Education and operation time were excluded from the binary logistic regression (see Table 2).

**Table 2:** Multicollinearity analysis (*n* = 505).

Variables	Tolerance	VIF
Age (years)	0.716	1.397
Gender	0.911	1.098
Type of surgery	0.505	1.979
**Education**	**0.213**	**4.700**
Stroke history	0.877	1.140
Coronary heart disease	0.907	1.103
ASA grade	0.906	1.104
ALB (g/L)	0.921	1.085
CRP (mg/L)	0.826	1.211
ALT (U/L)	0.953	1.049
GFR (ml/min)	0.892	1.121
Intraoperative blood loss (ml)	0.554	1.805
**Operation time (min)**	**0.232**	**4.311**
**Anesthesia time (min)**	**0.241**	**4.145**
**MOCA score (score)**	**0.206**	**4.864**
Weakened	0.813	1.230
Depression	0.885	1.130
Nutritional risk	0.841	1.189
Sleep disorder	0.937	1.068

Bold values indicate collinearity.

### 3.3 Binary logistic regression analysis of factors influencing dNCR after major orthopedic surgery in older adult patients

Binary logistic regression analysis was performed with postoperative dNCR occurrence as the dependent variable and variables with *p* < 0.2 in the univariate analysis as independent variables. Variable assignments are presented in Table 3. The logistic regression results showed that age, sleep disorder, anesthesia time, and stroke history were independent risk factors for postoperative dNCR, while the MoCA score was a protective factor (*p* < 0.05) (see Table 4). The Hosmer-Lemeshow test yielded χ^2^ = 6.475, *p* = 0.594 (*p* > 0.05), indicating a good fit of the regression model.

**Table 3:** Independent variable assignments (*n* = 505).

Variables	Value assignment
Age (years)	Use original values
Gender	Male = 0, Female = 1
Type of surgery	TKA = 0, THA = 1, HF = 2
Stroke history	No = 0, Yes = 1
Coronary heart disease	No = 0, Yes = 1
ASA grade	II = 0, III = 1
ALB (g/L)	Use original values
CRP (mg/L)	Use original values
ALT (U/L)	Use original values
GFR (ml/min)	Use original values
Intraoperative blood loss (ml)	Use original values
Anesthesia time (min)	Use original values
MOCA score (score)	Use original values
Weakened	No = 0, Yes = 1
Depression	No = 0, Yes = 1
Nutritional risk	No = 0, Yes = 1
Sleep disorder	No = 0, Yes = 1

**Table 4:** Binary logistic regression results (*n* = 505).

Variables	Coefficient	Wald	*P*	OR	LCL	UCL
**Age (years)**	**0.169**	**38.422**	**<0.001**	**1.184**	**1.123**	**1.249**
Gender (1)	0.469	2.125	0.145	1.598	0.851	3.002
Type of surgery		3.026	0.220			
Type of surgery (1)	0.634	2.47	0.116	1.886	0.855	4.159
Type of surgery (2)	0.674	1.67	0.196	1.962	0.706	5.455
**Stroke history (1)**	**1.021**	**4.15**	**0.042**	**2.776**	**1.039**	**7.414**
Coronary heart disease (1)	0.303	0.781	0.377	1.355	0.691	2.655
ASA (1)	0.195	0.297	0.586	1.215	0.603	2.449
ALB (g/L)	−0.028	0.952	0.329	0.972	0.919	1.029
CRP (mg/L)	−0.021	3.795	0.051	0.979	0.959	1.000
ALT (U/L)	0.004	0.134	0.714	1.004	0.984	1.024
GFR (ml/min)	−0.011	1.144	0.285	0.989	0.969	1.009
Intraoperative blood loss (ml)	−0.002	1.58	0.209	0.998	0.994	1.001
**Anesthesia time (min)**	**0.026**	**23.498**	**<0.001**	**1.026**	**1.015**	**1.037**
**MOCA score (score)**	**−0.221**	**27.169**	**<0.001**	**0.802**	**0.738**	**0.871**
Weakened (1)	0.568	2.000	0.157	1.765	0.803	3.881
Depression (1)	0.638	2.455	0.117	1.892	0.852	4.203
Nutritional risk (1)	−0.14	0.035	0.851	0.869	0.201	3.750
**Sleep disorder (1)**	**0.671**	**6.246**	**0.012**	**1.956**	**1.156**	**3.309**
Constant	−10.488	12.795	<0.001	0.000		

Bold values indicate *p* < 0.05.

### 3.4 Predictive contributions of various factors to dNCR after major orthopedic surgery in older adult patients

**Figure 2:**
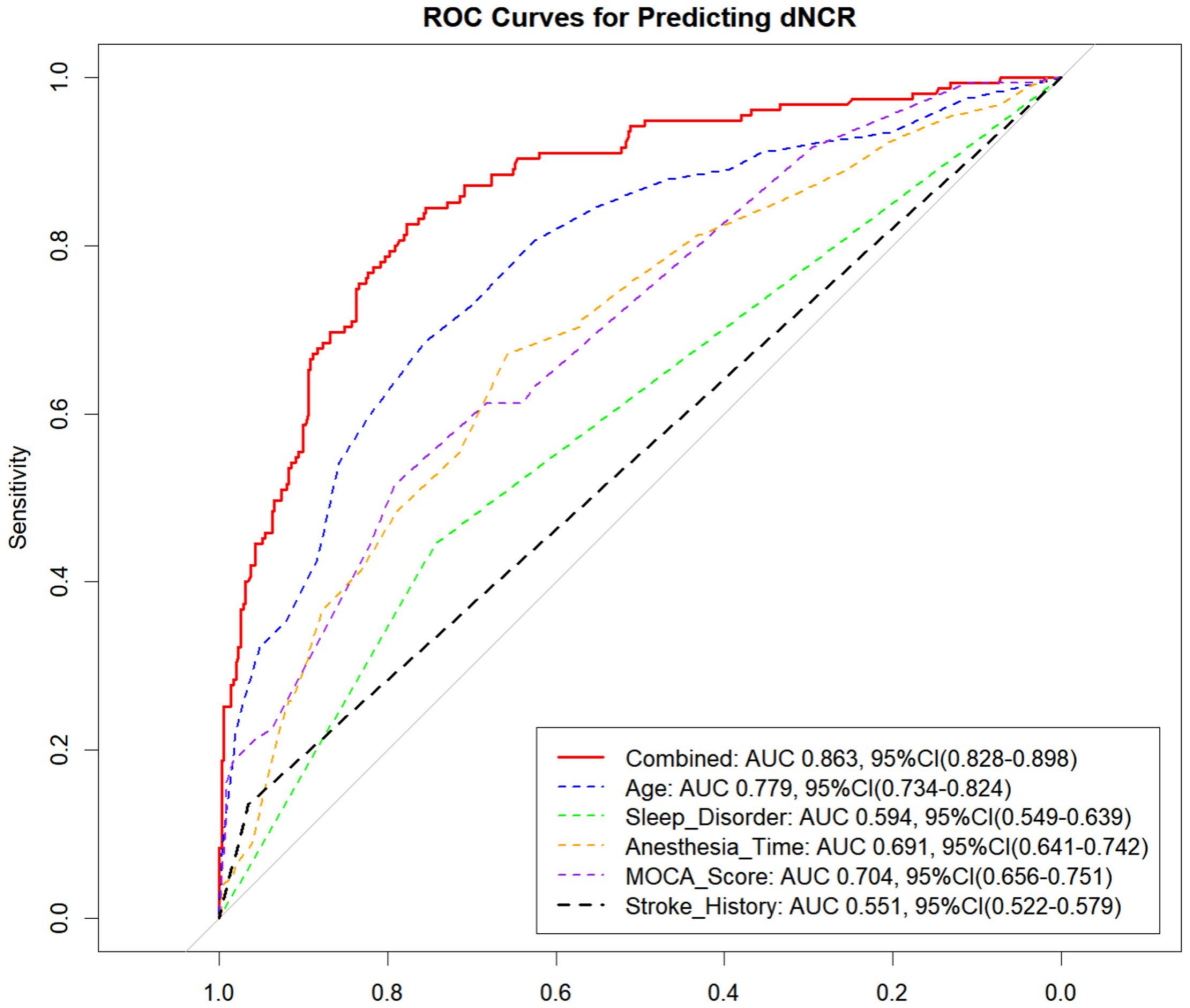
ROC curve (*n* = 505).

To further explore the individual and combined predictive value of each factor for dNCR, receiver operating characteristic (ROC) curves were plotted. For predicting postoperative dNCR, the area under the curve (AUC) of age was 0.779 (95% CI 0.734–0.824), sleep disorder was 0.594 (95% CI 0.549–0.639), and anesthesia time was 0.691 (95% CI 0.641–0.742). MoCA score was 0.704 (95% CI 0.656–0.751), and stroke history was 0.551 (95% CI 0.522–0.579). The combined prediction AUC for postoperative dNCR was 0.863 (95% CI 0.828–0.898). The corresponding ROC curves are shown in Figure 2, and the AUC comparisons by DeLong test are presented in Table 5.

**Table 5:** AUC comparisons by DeLong test (*n* = 505).

Variable	AUC	LCL	UCL	*Z*	*P*
Combined	0.863	0.828	0.898	NA	NA
Age	0.779	0.734	0.824	4.939	<0.001
Anesthesia time	0.691	0.641	0.742	6.919	<0.001
MOCA score	0.704	0.656	0.751	6.795	<0.001
Sleep disorder	0.594	0.549	0.639	10.040	<0.001
Stroke history	0.551	0.522	0.579	14.865	<0.001

*P* < 0.05; *P* were Bonferroni adjusted for multiple comparisons.

### 3.5 Handling of missing data

Of the 530 enrolled patients, 25 (4.7%) were lost to follow up. Baseline characteristics were similar between the lost to follow up and completed follow up groups (*p* > 0.05) (Supplementary Table 1). The coding of independent variables in the multiple imputation cohort is presented in Supplementary Table 2. The multivariable logistic regression results based on the multiple imputation cohort are shown in Table 3, which were consistent with those from the complete case analysis, suggesting that the findings were robust.

## 4 Discussion

This study showed that the overall incidence of early dNCR after major orthopedic surgery under general anesthesia in older adult patients was 30.69%, which is similar to the 31.36% reported after joint arthroplasty (30). However, our cohort included three surgical types with markedly different incidences: TKA 27.35%, THA 28.72%, and HF 46.67%. The incidence in HF patients was approximately twice that in TKA and THA patients, a trend consistent with previous findings that hip fracture carries a higher risk of postoperative cognitive decline (31, 32). Since HF accounted for only 11.9% (60/505) of our sample, the overall incidence was weighted by the lower rates in TKA and THA and thus closer to that of elective arthroplasty. This difference indicates that surgical type is an important factor influencing the incidence of dNCR. Furthermore, variations in assessment time windows, cognitive tools, diagnostic criteria for dNCR, and patient characteristics across studies may also lead to different incidence rates (33, 34).

After clarifying the incidence patterns, this study further identified independent factors associated with dNCR, including age, sleep disorder, anesthesia time, preoperative MoCA score, and stroke history.

Advanced age has been clearly identified as an independent risk factor for postoperative dNCR (35). Due to physiological function decline, older adult patients often suffer from chronic diseases, with the brain remaining in a chronic low-grade inflammatory state. These factors jointly accelerate cerebral white matter functional deterioration, manifesting as increased brain frailty (36). Additionally, decreased liver metabolic capacity in advanced age patients prolongs anesthetic drug action time, thus contributing to the high postoperative dNCR incidence in older adult patients (37). The above mechanisms suggest the necessity for a comprehensive assessment of older adult patients’ physical status, strengthening the nursing care of chronic diseases, and improving their nutritional status. Furthermore, early postoperative ambulation and cognitive training should be encouraged to strengthen the body’s metabolic capacity and brain cognitive reserve, thereby enhancing compensatory ability against surgical and anesthetic stress.

Sleep disorders have been identified as an important influencing factor for dNCR after surgery. Although the incidence of perioperative sleep disorders is as high as 60%, this issue has long been underappreciated by the medical community, and most patients have not actively sought medical help (38). Previous studies have shown a consistent association between sleep disorders and declines in cognitive domains such as executive function. The underlying mechanism may be that sleep is a critical period for memory consolidation, and sleep disruption interrupts this normal process (39). Therefore, timely interventions should be implemented for patients with preoperative sleep disorders. Among these, cognitive behavioral therapy for insomnia is regarded as the first-line non-pharmacological treatment, which can be supplemented with regular physical exercise (38). When necessary, pharmacological treatment may also be combined to achieve the best functional status for surgery.

Prolonged anesthesia duration is an influencing factor for postoperative dNCR. Anesthetic drugs directly act on the brain, inhibiting neural activity and potentially blocking central nervous system transmission. Previous studies have indicated that anesthetics affect amyloid beta and tau proteins, both directly related to cognition in the brain, thereby causing postoperative cognitive decline (40). For patients with an anticipated prolonged anesthesia duration, preoperative protective medication can be administered, such as dexmedetomidine (41) and intranasal insulin (42), which can lower the anesthetic drug threshold, reduce neurotoxicity, and alleviate peripheral inflammation. Meanwhile, strengthening surgical staff skill training and coordination can optimize anesthesia duration and surgical efficiency.

The MoCA score reflects preoperative cognitive status in patients; lower MoCA scores indicate worse cognitive function, which may increase the risk of postoperative dNCR. The cognitive reserve theory proposes that cognitive reserve plays a moderating and buffering role between pathological damage and clinical manifestations (43). Under the same pathological burden, high cognitive reserve individuals can rely on richer neural compensatory networks to maintain relatively better functional performance. Previous research indicates that patients with preoperative cognitive impairment have a nearly doubled probability of postoperative dNCR (44). In the present study, preoperative MoCA score was a protective factor against postoperative dNCR, further suggesting that routine preoperative cognitive screening should be strengthened. For patients with low preoperative MoCA scores, early cognitive training should be implemented. Currently developed cognitive function training includes neuropsychological games such as chain idiom solitaire, picture recognition, and number games. Additionally, mindfulness-based intervention and music therapy can be combined to provide inpatient and home-based cognitive training, thereby maintaining good preoperative cognitive reserve (45).

Stroke history is a risk factor for postoperative dNCR. Patients with stroke history have reduced tolerance to surgery and anesthesia, and sustained perioperative stress may readily further induce neurological injury, thereby increasing the risk of postoperative dNCR (46). In older adult patients, stroke history and advanced age can produce an additive effect, further raising the incidence of postoperative dNCR (47). Due to the inherent vulnerability and susceptibility of the nervous system in these patients, early preoperative cognitive prehabilitation should be initiated, including training in cognitive domains such as orientation, attention, and executive function (48). At the same time, emphasis should also be placed on overall preoperative physical exercise and daily living care, so as to improve patient outcomes.

However, the occurrence of postoperative dNCR in older adult patients is complex and influenced by multiple factors. Advanced age reduces cerebrovascular autoregulation. Older adult patients often have sleep disorders, such as shortened total sleep time, sleep fragmentation, and difficulty falling asleep. Sleep duration less than 6 h or more than 9 h significantly increases the risk of postoperative dNCR. These two factors, aging and sleep disorders, may act synergistically to compromise the brain’s tolerance to perioperative stress (49). Furthermore, prior stroke history causes organic neurological damage that exacerbates cerebrovascular dysfunction. This makes patients more vulnerable to surgical trauma and anesthetics, potentiating the neurotoxic effects of prolonged anesthesia exposure (50). Meanwhile, a low preoperative MoCA score reflects depleted baseline cognitive reserve, leaving the brain with insufficient compensatory capacity to withstand additional postoperative insults. The interaction of these factors imposes a multiple-hit injury on the nervous system through shared pathological pathways, including cerebral hypoperfusion, neuroinflammation, and impaired synaptic plasticity. These mechanisms ultimately lead to an elevated risk of postoperative dNCR. Therefore, clinicians should implement comprehensive perioperative risk assessments to enable early identification of high-risk patients. Active interventions should target modifiable factors, such as optimizing anesthetic management, improving sleep quality, and maintaining hemodynamic stability, thereby improving long-term patient outcomes.

## 5 Conclusion

In the present study, the incidence of postoperative early dNCR in older adult patients undergoing major orthopedic surgery under general anesthesia was 30.69%, which is relatively high. The development of postoperative dNCR is affected by multiple factors. Advanced age, sleep disorder, anesthesia time, and stroke history were identified as independent risk factors, while preoperative MoCA score was a protective factor. Based on these findings, clinical workers should develop individualized intervention strategies targeting the above factors to reduce the incidence of postoperative early dNCR and improve patient prognosis.

### 5.1 Limitations

Several limitations exist in this study. (1) Repeated postoperative MoCA assessments may be affected by multiple factors and introduce learning effects. Although this study used a relatively large threshold for the dNCR definition (a decrease of ≥1 standard deviation), which can partially reduce the interference of minor confounding factors and learning effects, it cannot completely exclude the influence of these factors on the results. Future studies should explore better methods to control for confounding factors. (2) All participants were recruited from a single tertiary hospital in Qingdao, which may lead to regional bias and restricted generalizability of the findings. Further multicenter studies are warranted to validate the present results and enhance their extrapolation to broader populations.

## Data Availability

The raw data supporting the conclusions of this article will be made available by the authors, without undue reservation.
