# Supplementary material for: Current status and influencing factors of early delayed neurocognitive recovery in older adult patients after major orthopedic surgery under general anesthesia: a cohort study
Source: Front Public Health. 2026 Aug 3;14:1887947. doi: 10.3389/fpubh.2026.1887947 (PMC13479793; doi:10.3389/fpubh.2026.1887947)
Supplement: Supplementary file 1 [file Supplementary_file_1.docx]

**Supplementary materials**

eTable 1: Baseline characteristics of the cohort by follow-up status

Table 2: Coding of independent variables in the multiple imputation cohort (*n*=530)

eTable 3: Multivariable logistic regression for dNCR using multiple imputation (*n*=530)

**eTable 1:** Baseline characteristics of the cohort by follow-up status

| Variable | Complete (*n*=505) | Lost (*n*=25) | *Z/χ^2^* | *P* |
| --- | --- | --- | --- | --- |
| Age | 70.00 (65.00, 74.00) | 69.00 (63.00, 73.00) | 0.223 | 0.824 |
| Sex | 381 (75.4%) | 21 (84.0%) | 0.542 | 0.462 |
| BMI | 25.89 (23.53, 28.65) | 25.35 (23.19, 27.11) | 1.153 | 0.249 |
| Education |  |  | 1.311 | 0.142 |
| Illiteracy | 72 (14.3%) | 1 (4.0%) |  |  |
| Primary school | 154 (30.5%) | 7 (28.0%) |  |  |
| Junior high school and above | 279 (55.2%) | 17 (68.0%) |  |  |
| Smoking | 48 (9.5%) | 4 (16.0%) | Fisher | 0.294 |
| Alcohol | 56 (11.1%) | 4 (16.0%) | Fisher | 0.511 |
| Preoperative MoCA | 25.00 (20.00, 25.00) | 25.00 (20.00, 26.00) | 0.908 | 0.354 |
| Type of surgery |  |  | 0.925 | 0.630 |
| TKA | 351(69.5%) | 18(72.0%) |  |  |
| THA | 94(18.6%) | 3(12.0%) |  |  |
| HF | 60(11.9%) | 4(16.0%) |  |  |
| NLR | 2.07 (1.51, 3.17) | 2.23 (1.70, 3.00) | 0.372 | 0.710 |
| CRP | 0.750 (0.500, 2.600) | 1.040 (0.500, 2.090) | 0.737 | 0.447 |
| ALT | 16.00 (12.00, 21.00) | 17.00 (12.00, 19.00) | 0.395 | 0.693 |
| AST | 19.00 (16.00, 23.00) | 18.00 (16.00, 24.00) | 0.437 | 0.662 |
| Cr | 53.00 (46.00, 63.00) | 47.00 (44.00, 58.00) | 1.531 | 0.126 |
| GFR | 94.48 (88.11, 100.27) | 95.43 (89.35, 99.70) | 0.477 | 0.634 |
| Intraoperative Blood Loss | 50.00 (50.00, 200.00) | 100.00 (50.00, 200.00) | 1.220 | 0.175 |
| Operation Time | 80.00 (70.00, 105.00) | 80.00 (65.00, 100.00) | 0.789 | 0.430 |
| Anesthesia Time | 150.00 (130.00, 165.00) | 155.00 (135.00, 170.00) | 0.379 | 0.705 |
| ALB | 42.80 (39.30, 44.80) | 43.00 (37.30, 44.30) | 0.650 | 0.516 |
| Weakened | 56 (11.1%) | 4 (16.0%) | Fisher | 0.511 |
| Depression | 57 (11.3%) | 4 (16.0%) | Fisher | 0.515 |
| ASA Grade | 407 (80.6%) | 19 (76.0%) | Fisher | 0.605 |
| Nutritional Risk | 18 (3.6%) | 2 (8.0%) | Fisher | 0.242 |
| Sleep Disorder | 159 (31.5%) | 9 (36.0%) | 0.064 | 0.800 |
| Stroke History | 33 (6.5%) | 2 (8.0%) | Fisher | 0.677 |
| Coronary Heart Disease | 75 (14.9%) | 4 (16.0%) | Fisher | 0.778 |
| Hypertension | 231 (45.7%) | 15 (60.0%) | 1.416 | 0.234 |
| Diabetes | 96 (19.0%) | 6 (24.0%) | Fisher | 0.602 |

**Table 2** Coding of independent variables in the multiple imputation cohort (*n*=530)

| **Variables** | **Value Assignment** |
| --- | --- |
| Age (years) | Use original values |
| Gender | Male=0,Female=1 |
| Stroke History | No = 0, Yes = 1 |
| Coronary Heart Disease | No = 0, Yes = 1 |
| ASA Grade | II = 0, III = 1 |
| ALB (g/L) | Use original values |
| CRP (mg/L) | Use original values |
| GFR (ml/min) | Use original values |
| Anesthesia Time (min) | Use original values |
| MOCA Score (score) | Use original values |
| Weakened | No = 0, Yes = 1 |
| Depression | No = 0, Yes = 1 |
| Nutritional Risk | No = 0, Yes = 1 |
| Sleep Disorder | No = 0, Yes = 1 |

**eTable 3:** Multivariable logistic regression for dNCR using multiple imputation (*n*=530)

| Variables | Coefficient | Wald | *P* | *OR* | *LCL* | *UCL* |
| --- | --- | --- | --- | --- | --- | --- |
| **Age (years)** | **0.171** | **40.048** | **<0.001** | **1.186** | **1.125** | **1.251** |
| Gender (1) | 0.465 | 2.184 | 0.140 | 1.592 | 0.858 | 2.954 |
| **Stroke History (1)** | **0.996** | **4.144** | **0.042** | **2.707** | **1.035** | **7.081** |
| Coronary Heart Disease (1) | 0.241 | 0.514 | 0.474 | 1.273 | 0.657 | 2.464 |
| ASA (1) | 0.254 | 0.548 | 0.460 | 1.290 | 0.656 | 2.535 |
| ALB (g/L) | -0.024 | 0.793 | 0.374 | 0.976 | 0.925 | 1.030 |
| CRP (mg/L) | -0.016 | 2.429 | 0.120 | 0.984 | 0.965 | 1.004 |
| GFR (ml/min) | -0.007 | 0.362 | 0.549 | 0.993 | 0.971 | 1.016 |
| **Anesthesia Time (min)** | **0.024** | **23.328** | **<0.001** | **1.024** | **1.014** | **1.034** |
| **MOCA Score (score)** | **-0.200** | **23.440** | **<0.001** | **0.819** | **0.755** | **0.888** |
| Weakened (1) | 0.544 | 2.055 | 0.152 | 1.722 | 0.817 | 3.628 |
| Depression (1) | 0.671 | 2.770 | 0.097 | 1.955 | 0.886 | 4.317 |
| Nutritional Risk (1) | -0.233 | 0.103 | 0.748 | 0.792 | 0.190 | 3.305 |
| **Sleep Disorder (1)** | **0.690** | **6.836** | **0.009** | **1.993** | **1.187** | **3.347** |
